# Large-Scale Production of *Cronobacter sakazakii* Bacteriophage Φ CS01 in Bioreactors via a Two-Stage Self-Cycling Process

**DOI:** 10.4014/jmb.2107.07017

**Published:** 2021-08-25

**Authors:** Jin-Sun Lee, Gyeong-Hwuii Kim, Jaegon Kim, Tae-Hyun Lim, Yong Won Yoon, Sung-Sik Yoon

**Affiliations:** Department of Biological Science and Technology, Yonsei University, Wonju 26493, Republic of Korea

**Keywords:** Bacteriophages, high cell density cultivation, *Cronobacter sakazakii*, two-stage self-cycling process, jar-type bioreactor, food safety

## Abstract

*Cronobacter sakazakii* is an opportunistic pathogenic bacterium found in powdered infant formula and is fatal to neonates. Antibiotic resistance has emerged owing to overuse of antibiotics. Therefore, demand for high-yield bacteriophages as an alternative to antibiotics has increased. Accordingly, we developed a modified mass-production method for bacteriophages by introducing a two-stage self-cycling (TSSC) process, which yielded high-concentration bacteriophage solutions by replenishing the nutritional medium at the beginning of each process, without additional challenge. pH of the culture medium was monitored in real-time during *C. sakazakii* growth and bacteriophage CS01 propagation, and the changes in various parameters were assessed. The pH of the culture medium dropped to 5.8 when the host bacteria reached the early log phase (OD_540_ = 0.3). After challenge, it decreased to 4.65 and then recovered to 4.94; therefore, we set the optimum pH to challenge the phage at 5.8 and that to harvest the phage at 4.94. We then compared phage production during the TSSC process in jar-type bioreactors and the batch culture process in shaker flasks. In the same volume of LB medium, the concentration of the phage titer solution obtained with the TSSC process was 24 times higher than that obtained with the batch culture process. Moreover, we stably obtained high concentrations of bacteriophage solutions for three cycles with the TSSC process. Overall, this modified TSSC process could simplify large-scale production of bacteriophage CS01 and reduce the unit cost of phage titer solution. These results could contribute to curing infants infected with antibiotic-resistant *C. sakazakii*.

## Introduction

In the 21st century, despite improvements in food hygiene, many children still die owing to meningitis caused by ingestion of powdered infant formula (PIF) infected with *Cronobacter sakazakii* [1]. *C. sakazakii* (previously known as *Enterobacter sakazakii*) is an opportunistic gram-negative, rod-shaped, pathogenic bacterium that is found in processed foods, including food products consumed daily, and arid environments, such as PIF [2]. *C. sakazakii* can easily contaminate PIF following improper storage, and consumption of this bacterium by neonates can cause meningitis and necrotizing enterocolitis because of their weak immune systems [3].

To prevent pathogen infection, extensive antibiotic administration has been used, resulting in the emergence of antibiotic-resistant bacteria, which has become a major problem worldwide [4]. Bacteriophages are alternatives to antibiotics that may be used to suppress antibiotic resistance and inhibit pathogen growth [5]. There are several advantages to using bacteriophages as therapeutic agents. First, unlike antibiotics, which act universally against various pathogens, bacteriophages can selectively kill pathogenic bacteria without killing beneficial bacteria because phages exhibit high host specificity [6]. Second, phages have therapeutic effects on antibiotic-resistant bacteria and are relatively safe to use. For example, since the 1930s, the Republic of Georgia in the Russian Federation has produced phages with clinical applications in the treatment of patients with antibiotic resistance [7]. Moreover, in 2006, the United States Food and Drug Administration permitted the use of purified bacteriophages as food additives [8]. Third, it is not difficult to obtain host-specific phages because they often coexist in the host bacteria living environment. For example, phage CS01 was isolated by Kim *et al*. from swine feces containing *C. sakazakii* [9]. Based on these strengths, bacteriophages are utilized in many fields, including the medical and food industries [10]. However, efficient methods for large-scale production of bacteriophages are limited. The batch-culture method using flasks is insufficient for satisfying the demands of using bacteriophages commercially. The batch-culture process is not a continuous process but a single process in which the culture conditions, except temperature and rpm, cannot be regulated. Thus, it is difficult to lower production costs using this approach owing to the low production efficiency of the method.

**Fig. 1:**
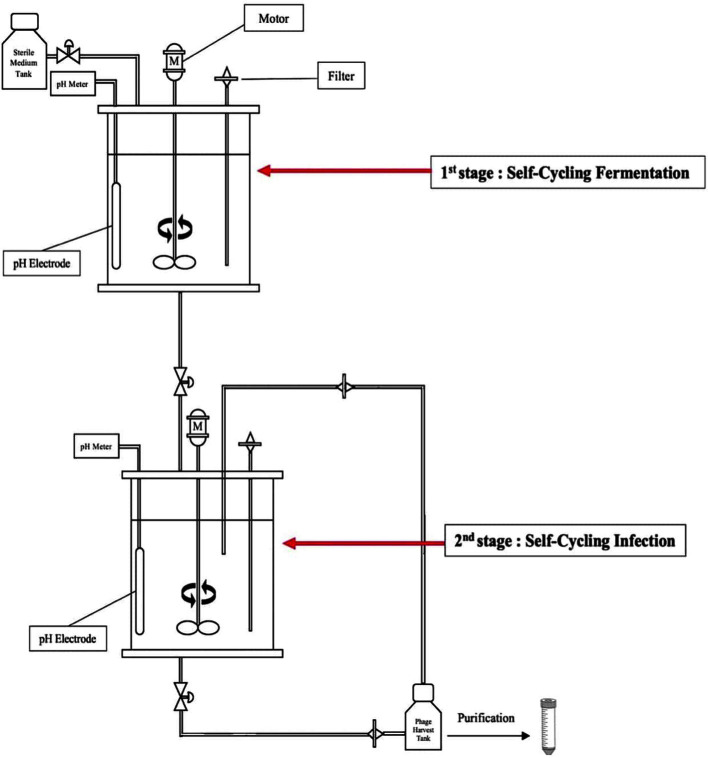
Schematic diagram of the TSSC process.

In this study, we introduced a semicontinuous two-stage self-cycling (TSSC) process [11] for the mass-production of large quantities of bacteriophages using jar-type bioreactors which included a self-cycling fermentation (SCF) stage and self-cycling infection (SCI) stage (Fig. 1A). Using the TSSC process, we continually obtained high-concentration phage titer solutions by adding nutrient medium at the beginning of the process, without additional challenge. The titers showed good activity against *C. sakazakii* and demonstrated increased efficiency. Overall, our approach using the TSSC process could have important applications in achieving phage yields higher than those obtained using the traditional batch culture system.

## Materials and Methods

### Bacteriophage Strain

The bacteriophage Φ CS01 used in this study was obtained from Kim *et al*. and had been isolated from swine feces obtained from a pig farm located in Gangwon Province, Republic of Korea. In a previous study, the phage was shown to retain most of its infectivity after 1 h of incubation at 4–37°C. However, the infectivity of the phage was slightly reduced at 50°C and 60°C and completely abolished at 70°C. Additionally, in the pH stability test, there was no reduction in the infectivity of the phage after 1 h of incubation at pH 4–9. However, the infectivity was reduced at pH 10–11 and no infectivity was observed at pH 3 or 12 [9].

### Host Bacterial Strain and Growth Curve

The bacterium (*C. sakazakii* NCTC 11467) used in this experiment was obtained from the Korean Collection for Type Cultures (Korea). Bacterial cultures were adjusted to an optical density at 540 nm (OD_540_) of 0.1 using a microplate reader (Multiskan FC; Thermo, USA) and grown in TSB broth (BD Bacto, USA) under microaerophilic conditions in a shaking incubator at 37°C for 3 h after inoculating 20 μl/ml of the host bacteria. Colony forming units (CFUs) of cultured cells were measured every 10 min [12]. Analysis of the in vitro growth curves of *C. sakazakii* revealed that the lag phase was approximately 30 min and that 140 min was required to complete exponential growth after inoculation (logarithmic phase). Subsequently, cells entered the stationary phase. Furthermore, for continual circulation between the first and second stages, the temperature of the first jar fermentor was adjusted to 24°C to enable the host bacteria to reach an early log phase (OD_540_ = 0.3, pH = 5.8) after 4 h. Further experiments confirmed that except for the growth rate, there were no differences between incubating the host at 24°C and 37°C.

### One-Step Growth Curve of Bacteriophages

For one-step growth curve analysis, we centrifuged 5 ml bacterial culture in the exponential phase (OD_540_ = 0.3) at 4,000 ×*g* for 10 min. The pellet was resuspended in 1 ml TSB broth, and the phage solution was added to the suspension at a multiplicity of infection (MOI) of 0.1. The mixture was incubated at 37°C for 30 min and then centrifuged at 11,000 ×*g* for 10 min. The pellet was resuspended in 10 ml TSB broth, and the bacteriophage titer (PFU/ml) was measured at 10-min intervals for 120 min using double-layer plaque assays.

### Amplification of the Bacteriophage

A single colony of the host strain (*C. sakazakii*) was resuspended in 5 ml TSB and incubated at 37°C in a shaking incubator (compact shaking incubator, JSSI-100T; JSR) at 100 rpm. When the culture reached the early log phase (OD_540_ of 0.3), 1 ml solution was subcultured into 100 ml TSB broth, followed by incubation at 37°C in a shaking incubator at 100 rpm until the early log phase was reached (OD_540_ of 0.3). Phages were challenged at an MOI of 0.1 and cultivated for 20 min at 37°C in a shaking incubator in 100 rpm for 4 h [13].

### Purification of the Bacteriophage

After amplification of bacteriophages, 1 ml (1% w/v) chloroform and 1 M NaCl were added. Samples were then further incubated at 37°C in a shaking incubator at 200 rpm for 30 min. The culture was centrifuged at 11,000 ×*g* for 30 min at 4°C, and the supernatant was filtered using a syringe filter with a pore size of 0.45 μm (Advantec, USA). Solid polyethylene glycol (PEG 8000; Aldrich, USA) was added to the suspension to achieve a final concentration of 20% (w/v), and the mixture was cooled on ice overnight. The lysate was then centrifuged at 11,000 ×*g* for 100 min, the supernatant was discarded, and the pellet was resuspended in 5 ml SM buffer. Next, a half volume of chloroform was added, and samples were mixed gently, followed by centrifugation at 3,000 ×*g* for 15 min at 4°C. The supernatant was recovered, filtered using a syringe filter with a pore size of 0.45 μm (Advantec), and stored at 4°C in a refrigerator [14].

### Double Layer Plaque Assay

When spreading a suspension of an infective phage (phage Φ CS01) over the lawn of susceptible bacterial cells (*e.g.*, *C. sakazakii*), the phage attaches to the bacterial cell, replicates inside the bacterial cell, and kills the cell during lytic release. This forms a clearing zone called a plaque within the lawn of the bacteria. In the absence of the lytic phage, the bacteria form a confluent lawn of growth. Accordingly, we counted the number of plaques and used the dilution factors to calculate the PFU in a phage titer solution [15]. The PFU were determined using plaque-forming assays.

### One-Step Growth Curve of Bacteriophage

For one-step growth curve analysis, we centrifuged 5 ml bacterial culture in the exponential phase (OD_540_ = 0.3) at 4,000 ×*g* for 10 min. The pellet was resuspended in 1 ml TSB broth, and the phage solution was added to the suspension at an MOI of 0.1. The mixture was incubated at 37°C for 30 min and then centrifuged at 11,000 ×*g* for 10 min. The pellet was resuspended in 10 ml TSB broth, and the bacteriophage titer (PFU/ml) was measured at 10-min intervals for 120 min using the double-layer plaque assay [16].

### TSSC Process

The TSSC process consisted of a SCF stage and SCI stage. In each stage, independent Jar-type bioreactors (LiFlus GX; Hanil Scientific Inc., Korea) were used. To prevent contamination of bacteria other than *C. sakazakii* under aerophilic conditions, bioreactors, accessory equipment, and TSB broth were sterilized using an autoclave. When transferring the culture medium from the SCF stage to the SCI stage, sterilized plastic tubes and a peristaltic pump machine were used, and external air was introduced through a 0.45 μm membrane filter (Jet Biofil, USA). Additionally, during the sampling process, sterilization was maintained through flame sterilization.

To increase the productivity of the bacteriophage culture medium, we measured changes in various parameters (*i.e.*, pH, OD, viable cell count, and PFU) according to the growth of *C. sakazakii* and propagation of the bacteriophage.

The pH of the medium was measured in real-time using a pH meter installed in a Jar-type bioreactor (LiFlus GX; Hanil Scientific Inc.), and changes in the pH of the phage solution were used to determine the optimal challenge and harvest times of the phage culture medium. We did not adjust the pH of the culture medium when monitoring changes in the pH according to the growth and death of *C. sakazakii*.

Notably, during the growth of *C. sakazakii*, the pH of the culture medium decreased as the host grew because the host strain excreted acidic substances during growth. By contrast, after challenging the bacteria with bacteriophage Φ CS01, the pH recovered during phage propagation. We assumed that this result was observed because of release of the intracellular components from the lysed host bacteria, which could act as a buffer in the culture medium.

### SCF Stage

In the SCF stage, a single colony of the host strain was resuspended in 10 ml TSB broth and incubated at 37°C until the early log phase was reached (OD_540_ = 0.3, pH = 5.8). After cultivation, 1 ml culture solution was subcultured in the first jar fermentor with 1% v/v (10 ml in 1,000 ml host culture at an OD_540_ of 0.3) infection load. The bacterial culture was cultivated at 24°C and 100 rpm for 4 h until reaching early log phase (OD_540_ = 0.3, pH = 5.8), and this condition was adjusted to synchronize with the growth rate of phage CS01 in the SCI stage. After propagating the host bacteria, 900 ml culture medium was transferred to a second jar fermentor, and 100 ml solution remained. Immediately after transferring the culture medium, 900 ml fresh medium (TSB) was added to the first jar fermentor [17].

### SCI Stage

In the SCI stage, the bacteriophage culture was added to a second jar fermentor with an MOI of 0.1 and cultivated at 37°C and 100 rpm for 4 h. Temperature and rpm were adjusted to optimize the infectivity of phage CS01. After cultivation, when the pH of the lysate reached 5.0, the samples were harvested and filtered using a bottle-top vacuum filter with a pore size of 0.45 μm (Jet Biofil). After filtration, 900 ml filtered solution was purified with PEG, NaCl, and chloroform to yield a high-titer bacteriophage solution, and 100 ml filtered solution was added to a second jar fermentor for continual infection. The whole process was repeated three times [18].

### Establishment of Optimal Infection Conditions for Bacteriophages

Experiments were conducted to determine the optimal initial infection conditions for phage production. The infection loads tested ranged from 1% to 5% v/v (0.1–0.5 ml in 10-ml cultures) of host cultures at an OD_540_ of 0.4 (cell concentration of approximately 5.0 × 10^8^ CFU/ml), and the initial MOIs tested were 1.0 × 10^-4^ to 1.0 × 10^-1^. The bacteriophage titer was calculated as the PFU/ml of the bacteriophage solution, as measured using double-layer plaque assays [18].

### Transmission Electron Microscopy (TEM)

The morphology of phage CS01 was observed using TEM. Based on the International Committee guidelines on the Taxonomy of Viruses, we conducted identification and classification of phage CS01 [19]. A high concentration (10^9^ PFU) of phage CS01 solution was prepared using the phage amplification method described above. Subsequently, 10 μL phage CS01 titer solution was loaded on 200-mesh grids coated with a collodion film prepared from 2% collodion in amyl acetate and incubated for 1 min. Next, the same amount of 2% uranyl acetate was applied and washed with ultrapure water. The stained grid was subjected to TEM analysis with a JEM-2100F field emission transmission electron microscope (Jeol, Korea) [20].

### Statistical Analysis

The results are expressed as means ± standard deviations. Student’s *t*-tests were used to evaluate the significance of differences between groups. Results with *p* values less than 0.05 were considered significant. All analyses were performed using SPSS software (SPSS Inc., USA).

## Results

### One-Step Growth Curve of Phage Φ CS01

**Fig. 2:**
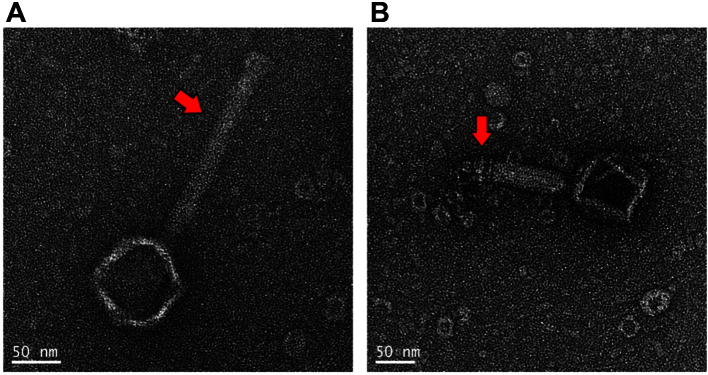
Transmission electron micrographs of *Cronobacter sakazakii* phage Φ CS01. Phage Φ CS01 is shown with an extended tail sheath (**A**) and contracted tail sheath (**B**), substantiating that it is a *Myoviridae* phage.

Analysis of the one-step growth curve of Φ CS01 (Fig. 2) revealed that the latent period was approximately 60 min after challenge, and the logarithmic period was from 60 to 80 min. Moreover, 80 min was required to complete the burst after initial infection with a burst size of 9.8 viral particles per host cell (PFU/infected cell).

### Optimal Initial Infection Conditions for Phage Φ CS01 Production

**Fig. 3:**
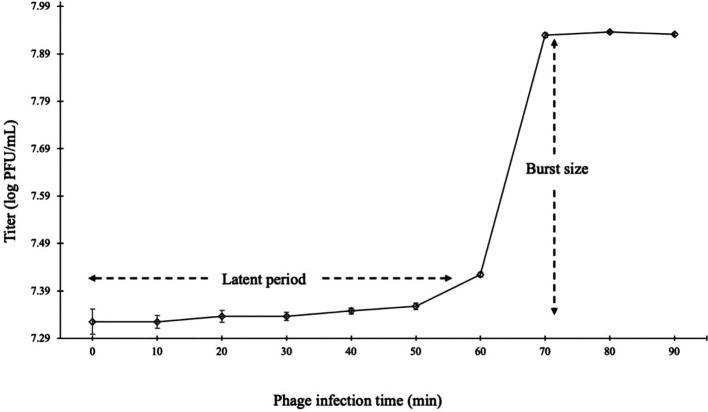
One-step growth curve of phage Φ CS01 on *Cronobacter sakazakii* NCTC 11467 at 37°C. The phage growth parameters are indicated in the figure, showing the latent period (60 min) and the average burst size (9.8 viral particles/ host cell).

We confirmed the optimal initial infection conditions for phage Φ CS01 (Fig. 3). The optimal titers were obtained from an infection load of 1% and an initial MOI between 10^-1^ and 10^-2^. Thus, we selected the following conditions for bioreactor operation: infection load, 1%; initial MOI, 10^-1^.

### Changes in pH, Viable Cell Counts, and OD of *C. sakazakii* Culture Medium

**Fig. 4:**
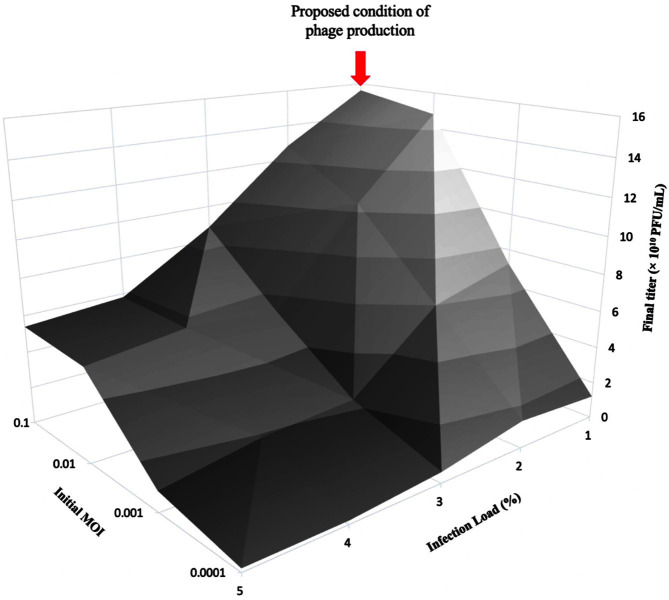
Optimal initial infection conditions for phage Φ CS01 production.

Fig. 4A shows changes in the viable cell counts and OD of the bacterial culture. Generally, there was a direct correlation between the viable cell count and OD. However, as shown in Fig. 4B, there was an inverse correlation between pH and OD. During cultivation, the pH of the culture medium decreased from 6.4 to 4.5 as the OD increased from 0.05 to 0.62. This result confirmed that the bacteria were in the logarithmic growth phase when the pH of the culture medium was approximately 5.8. Similarly, we observed an inverse correlation between pH and viable cell count in culture (Fig. 4C); *C. sakazakii* cultures were in the log phase when the pH of the culture medium was around 5.8.

### Bacterial Challenge Test of Phage Φ CS01 with *C. sakazakii*

**Fig. 5:**
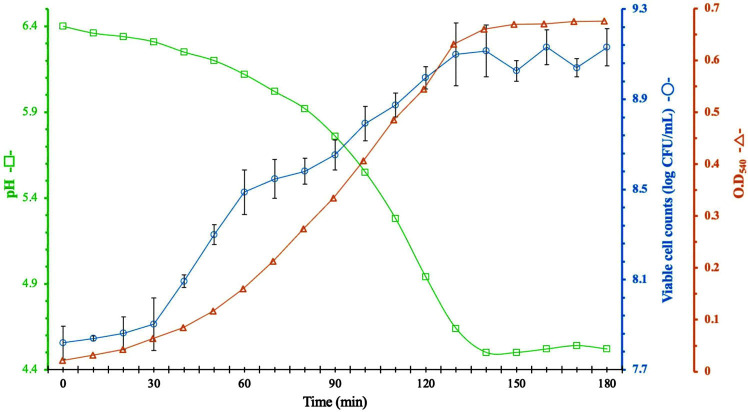
Variations in pH, viable cell count, and OD_540_ of bacterial culture during cultivation. The suspension of *Cronobacter sakazakii* NCTC 11467 was adjusted to an optical density at 540nm (OD_540_) of 0.1, followed by culture in TSB broth on a shaking incubator at 37°C. Changes in viable cell count (solid line with circles), OD_540_ (solid line with triangles), and pH (solid line with squares).

Next, we tested the effects of phage Φ CS01 on the growth of *C. sakazakii* after 180 min of cultivation at 37°C (Fig. 5). Notably, we observed differences in the viable cell counts and OD_540_ values between experimental and control groups in the late logarithmic phase (Figs. 5A and 5B). Moreover, the pH of the culture medium differed between the experimental and control groups in the late log phase (Fig. 5C). These results suggested that the lysis activity of the phage may explain the recovery of the pH of the culture medium.

### Characteristics of Phage Φ CS01 Infection in *C. sakazakii*

**Fig. 6:**
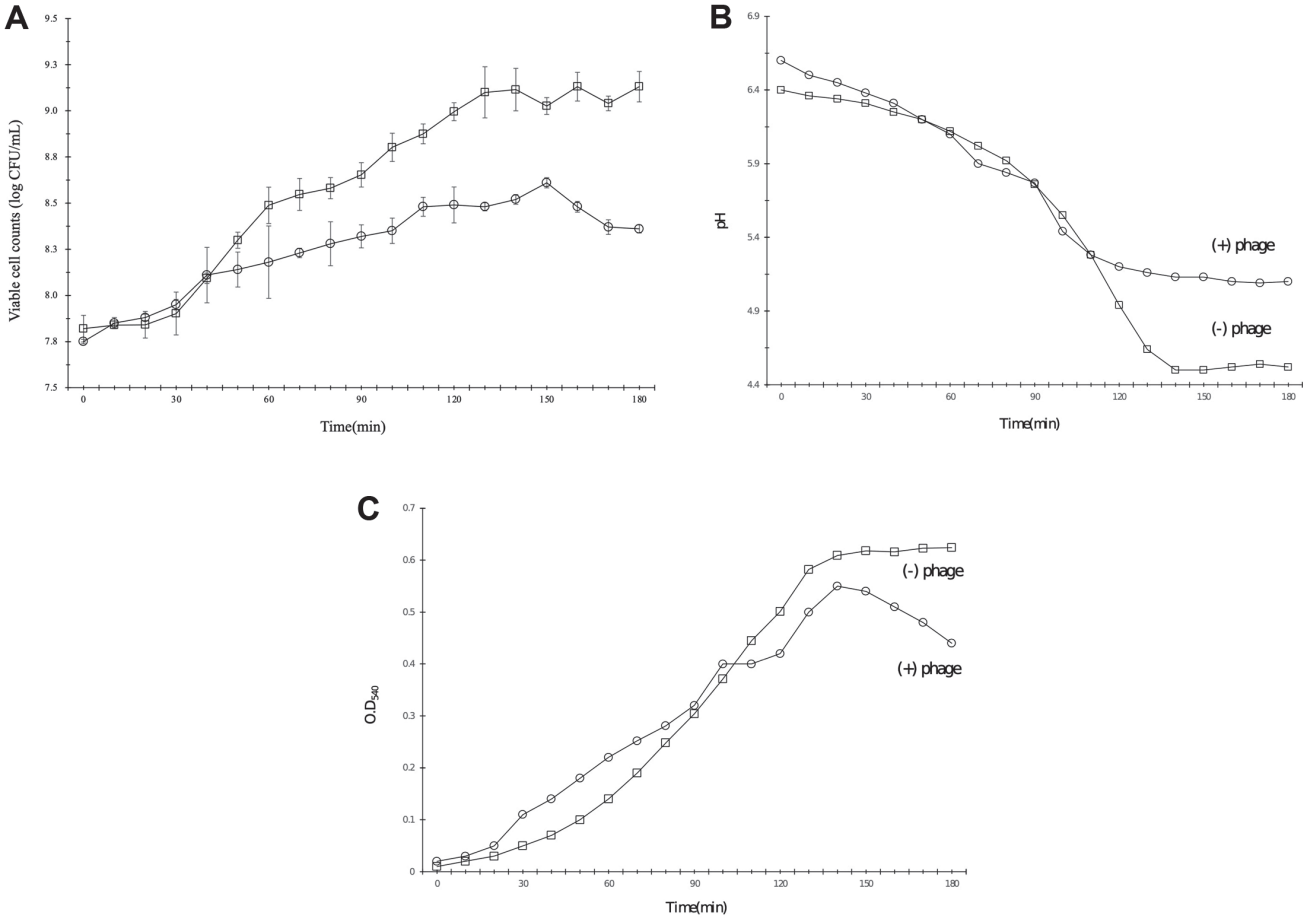
Bacterial challenge tests for phage Φ CS01 with *Cronobacter sakazakii* NCTC11467. The graphs show the viable cell count (**A**), OD_540_ (**B**), and pH (**C**) of the bacterial cultures. Measurements were made every 10 min. Phage Φ CS01 was used to challenge host bacteria when the OD of the bacterial culture at 540 nm was 0.1. Non-Φ CS01-infected culture is shown as a solid line with squares, and Φ CS01-infected culture is shown as a solid line with circles. The error bars represent the standard deviations of three determinations.

We then evaluated changes in the pH and titer of the bacterial culture after infection with the phage (Fig. 6). The pH of the culture medium was reduced from 5.61 to 4.65 at 60 min after challenge and then slowly recovered to 5.08. Additionally, the phage titer was slightly increased at 30 min after inoculation and then increased sharply. The results suggested that the phage caused bacterial cell lysis, resulting in the entry of intracellular substances into the culture medium, where they acted as a buffer in the medium; this led to recovery of the pH.

Notably, when the pH recovered after challenge with the phage (Fig. 6B), there was an inverse relationship between the pH and viable cell count. We assumed that the culture pH recovered as the viable cell count decreased owing to the lytic effects of the phage. Similarly, as shown in Fig. 6C, as the concentration of phage Φ CS01 increased, the viable cell count in the culture medium decreased.

### High-Titer Phage Yields with the TSSC Process

**Fig. 7:**
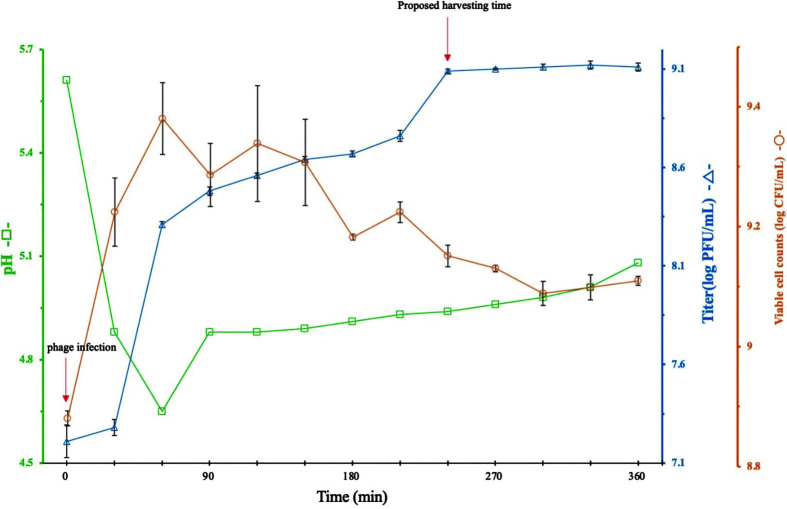
Characteristics of phage Φ CS01 challenge in *Cronobacter sakazakii* NCTC11467. The graphs show the viable cell count (solid line with circles), titers of phage Φ CS01 (solid line with triangles), and pH (solid line with squares). Measurements were made every 30 min. Phage Φ CS01 was used to challenge host bacteria when the OD of the bacterial culture at 540 nm was 0.3. The error bars represent the standard deviations of three determinations.

Finally, we evaluated the phage yields obtained using the TSSC process. Our results showed that TSSC with a jar-type bioreactor yielded 24-fold higher bacteriophage titers than that obtained using the conventional batch culture method. By conveniently adding the nutrient medium at the beginning of the process and without adding more phage solution or bacterial culture, we continually obtained high titers of the phage lysate, and these titers showed good activity against *C. sakazakii* (Fig. 7). The efficiency was increased because the culture environments of the host bacteria and bacteriophage were separated.

## Discussion

Propagating bacteriophage via the batch culture process typically ends with a single harvest and requires inoculation of additional host bacteria and challenge phage solution during each process. In a previous study, Kim *et al*. prepared high-titer phage solution of phage CS01 via the batch culture process in a small scale (500 ml). In order to improve large-scale phage production efficiency, we introduced the TSSC process, a method using two jar-type bioreactors, which can produce large quantities of phage titer solution without inoculating additional bacteria and challenging phage solution [11]. In addition, we focused on pH changes in culture medium during the TSSC process. Specifically, during the growth of *C. sakazakii*, the pH of the culture medium decreased as the host grew because the host strain excreted acidic substances during growth. By contrast, after challenging the bacteria with bacteriophage Φ CS01, the pH recovered during phage propagation. We assumed that this was caused by release of the intracellular components from the lysed host bacteria, which could act as a buffer in the culture medium. Considering this change, we established a modified TSSC process that could evaluate the optimal pH conditions for culturing Φ CS01 in real-time using a pH meter. As a result, the titer of the phage solution obtained with the TSSC process was 24 times higher than that obtained through the fed-batch method. Furthermore, during the third round of the process, high-titer phage solutions (5.0 × 10^10^ PFU/mL) were stably obtained.

However, there were some limitations to this study. First, we determined that the cause of pH rebound was related to the buffer action and the release of intracellular substances following host cell lysis. We deliberately induced cell lysis in *C. sakazakii* by adding ethylenediaminetetraacetic acid (EDTA) and lysozyme into actively growing host cells and sonicated the samples to verify this hypothesis [21]. Although this pH change was conclusively observed, we were not able to obtain definitive data demonstrating that this change was directly associated with cell lysis. This is because even if the pH of the EDTA stock solution was adjusted to the same pH as the culture medium prior to addition, the pH of the EDTA solution itself was 8.0, and this may have been an external factor also affecting the pH change. Therefore, further experiments are needed to address to this change in pH in our experiment. Second, because the TSSC process used in this experiment estimated the phase of the host bacteria through changes in pH, this method may only be applicable for bacterial strains, which show alterations in pH during growth.

In summary, the pH of the host culture medium dropped to 5.8 when the host bacteria reached the early log phase (OD_540_ = 0.3), and after challenge with phage Φ CS01, the pH decreased to 4.65 and then recovered to 4.94 after 4 h. There were no significant changes in the phage titer and pH after incubation for 4 h; therefore, we determined that the optimal incubation time was 4 h. Overall, our findings established that the TSSC process could simplify bacteriophage production. Moreover, we previously estimated the phase of the host bacteria by measuring the OD value of the culture medium. By implementing real-time pH measurement with a pH meter, we could simplify the process of producing bacteriophage. Additionally, because the pH meter measured the pH of the entire solution in real-time, this approach had the advantage of a relatively low error rate compared with measurement of the OD of a part of the culture medium. Thus, cultivation of the bacteriophage by applying a simplified continual cultivation process could facilitate commercialization of the bacteriophage by lowering the unit cost.

## Figures and Tables

**Fig. 8:**
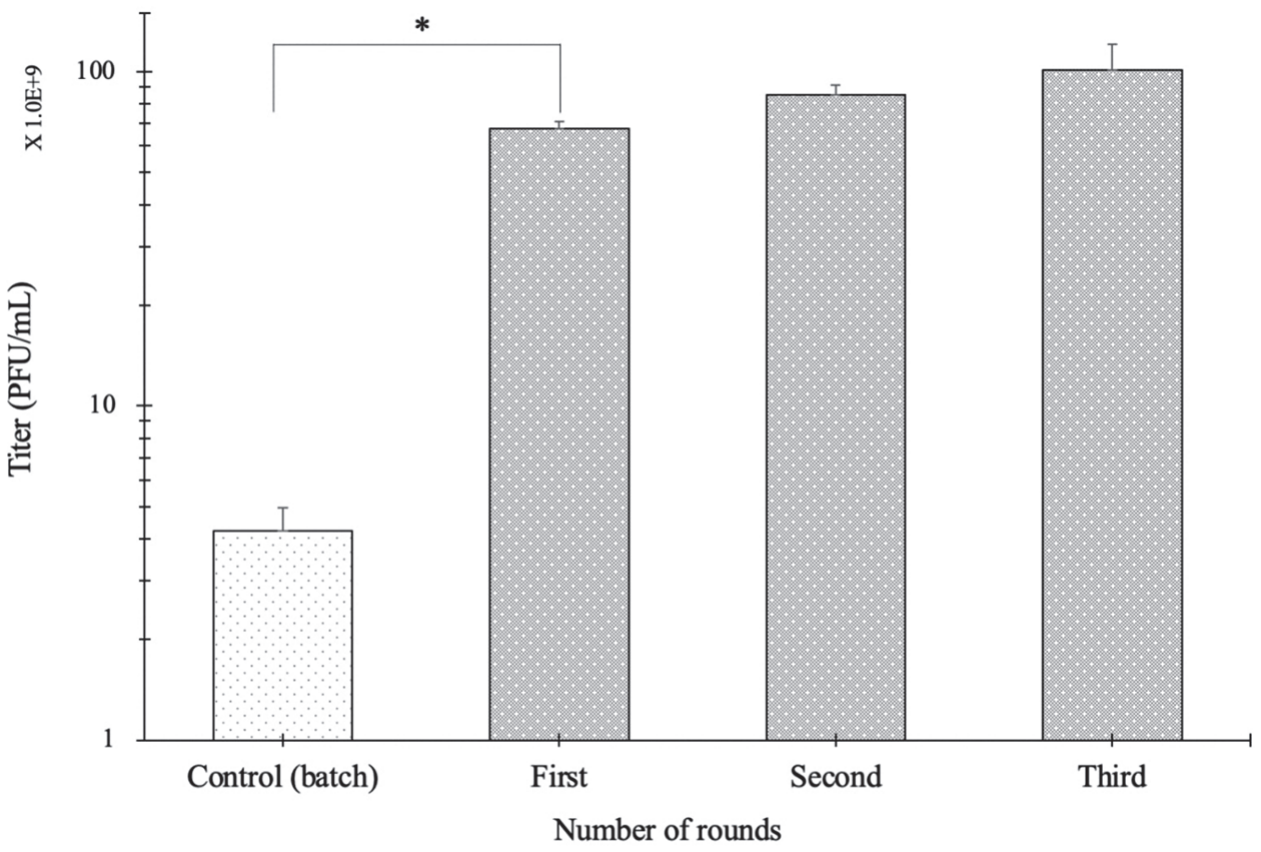
High-titer phage solutions were obtained by the third round of cultivation with the TSSC process. The error bars represent the standard deviations of three determinations. *p < 0.05.
